# IgG4-related retroperitoneal fibrosis mimicking perinephric extension of renal cell carcinoma on CT: a case report

**DOI:** 10.1093/bjrcr/uaag016

**Published:** 2026-04-20

**Authors:** Alvin J Logronio, Marta Prieto-Suárez, José Antonio Ortiz-Rey, Milagros Otero-García

**Affiliations:** Department of Medical Imaging and Therapeutic Radiology, National Kidney and Transplant Institute (NKTI), Quezon City, Metropolitan Manila, 1101, Philippines; Department of Pathology, Complejo Hospitalario Universitario de Vigo (CHUVI), Vigo, Galicia, 36312, Spain; Department of Pathology, Complejo Hospitalario Universitario de Vigo (CHUVI), Vigo, Galicia, 36312, Spain; Department of Radiology, Complejo Hospitalario Universitario de Vigo (CHUVI), Instituto de Investigación Sanitaria Galicia Sur (IISGS), Vigo, Galicia, 36312, Spain

**Keywords:** case report, IgG4-related disease, retroperitoneal fibrosis, renal cell carcinoma, perinephric fat, computed tomography, tumour staging, radiologic pitfall

## Abstract

Immunoglobulin G4-related disease (IgG4-RD) is an immune-mediated fibroinflammatory condition that can closely mimic malignancy on imaging. IgG4-related retroperitoneal fibrosis (RPF) most commonly involves the para-aortic or peri-iliac regions, while focal perinephric involvement is uncommon. Accurate differentiation from true perinephric tumour extension is critical, as misinterpretation may substantially alter renal cell carcinoma (RCC) staging and management. A 41-year-old man undergoing contrast-enhanced CT for transient rectal bleeding was incidentally found to have a heterogeneously enhancing left renal mass suspicious for RCC, with adjacent unilateral irregular perinephric soft tissue extending beyond Gerota’s fascia. On CT, these findings were interpreted as locally advanced cT4 RCC. Radical nephrectomy was performed. Histopathology demonstrated clear cell RCC, grade 2, confined to the kidney (pT1b), with intact renal capsule and no perinephric or lymphovascular invasion. The adjacent perinephric tissue instead showed storiform fibrosis, dense IgG4-positive plasma cell infiltrates, and obliterative phlebitis, consistent with IgG4-related RPF. Serum IgG4 was mildly elevated. Postoperative FDG PET/CT demonstrated a focal hypermetabolic perinephric lesion without systemic involvement, and both imaging and serologic findings improved following corticosteroid therapy. This case highlights a rare but important radiologic pitfall in which focal perinephric IgG4-related RPF was radiologically indistinguishable from perinephric RCC extension, resulting in initial overstaging. IgG4-RD should be considered in the differential diagnosis when perinephric soft tissue appears inseparable from a renal mass, and multimodal evaluation integrating imaging, pathology, and serology is essential to avoid overstaging and inappropriate management.

## Introduction

Immunoglobulin G4 (IgG4)-related disease (IgG4-RD) is an immune-mediated fibroinflammatory condition characterised by tumefactive lesions composed of dense lymphoplasmacytic infiltrates rich in IgG4-positive plasma cells, storiform (whorled) fibrosis, and obliterative phlebitis, often, but not invariably, associated with elevated serum IgG4 concentrations.^1^^,^^2^ Although initially described only in the pancreas, IgG4-RD has recently been recognised as a systemic clinicopathologic entity. It is now known to involve a broad spectrum of organs, including the pancreas, biliary tract, salivary and lacrimal glands, kidneys, lungs, aorta, retroperitoneum, lymph nodes, and others.^1^^,^^2^

From a radiologic standpoint, IgG4-RD is particularly challenging because of its protean imaging manifestations. Lesions frequently present as mass-like abnormalities, infiltrative soft-tissue thickening, or organ enlargement, often with imaging characteristics that closely resemble malignancy.^1^^,^^2^ On cross-sectional imaging, IgG4-RD may appear as a solid soft tissue lesion with variable enhancement, while functional imaging such as fluorine-18 fluorodeoxyglucose (^18^F-FDG) PET/CT often demonstrates avid tracer uptake reflecting active inflammation. These features, although helpful for detecting disease extent, significantly limit specificity and contribute to frequent diagnostic confusion.

IgG4-related retroperitoneal fibrosis (RPF) represents a recognised manifestation within the IgG4-RD spectrum and accounts for a substantial proportion of cases previously labelled as idiopathic retroperitoneal fibrosis.^1^^,^^2^ Classically, IgG4-related RPF manifests as plaque-like soft tissue encasing the abdominal aorta, iliac vessels, or ureters, often leading to obstructive uropathy. Perinephric involvement may occur but typically presents as circumferential soft-tissue thickening surrounding one or both kidneys rather than as a focal mass.^1^^,^^2^ Focal perinephric IgG4-related RPF is distinctly uncommon.

The perinephric region is also a critical anatomic compartment in renal cell carcinoma (RCC) staging. Imaging evidence of tumour extension into the perinephric fat upgrades disease to at least stage T3, while invasion beyond Gerota’s fascia corresponds to stage T4 disease.^3^ Accurate radiologic assessment of perinephric involvement is therefore essential, as overstaging may lead to more aggressive surgical management and preclude nephron-sparing approaches.

We report a rare case in which focal IgG4-related RPF closely mimicked perinephric extension of RCC on CT, resulting in radiologic overstaging as T4 disease. Histopathologic evaluation ultimately demonstrated two distinct processes: a pT1b clear cell RCC confined to the kidney and a separate focus of IgG4-related RPF immediately adjacent to it. IgG4-related fibroinflammatory lesions are recognised malignancy mimics and have been reported to lead to unnecessary surgical intervention when misinterpreted.^4–6^ Although coexistence of IgG4-RD and RCC and other malignancies has been described, prior reports have not emphasised a focal perinephric lesion producing a direct staging mimic.^7–9^

This case highlights an important diagnostic pitfall for radiologists and urologists alike. The combination of an enhancing renal mass and inseparable perinephric soft tissue without a clear intervening fat plane created a convincing imaging appearance of locally advanced RCC. Awareness of IgG4-RD as a potential inflammatory mimic in this setting is therefore critical to avoid overstaging and inappropriate management.

## Case presentation

### Patient information

A 41-year-old man with no significant prior medical history underwent cross-sectional abdominal imaging as part of an evaluation for recurrent rectal bleeding. He had no known chronic medical conditions, no relevant family history of malignancy or autoimmune disease, and was not taking long-term medications. He denied constitutional symptoms, flank pain, haematuria, or urinary complaints. The rectal bleeding resolved spontaneously and was ultimately unrelated to the rest of the clinical course.

### Clinical course

**Figure 1:**
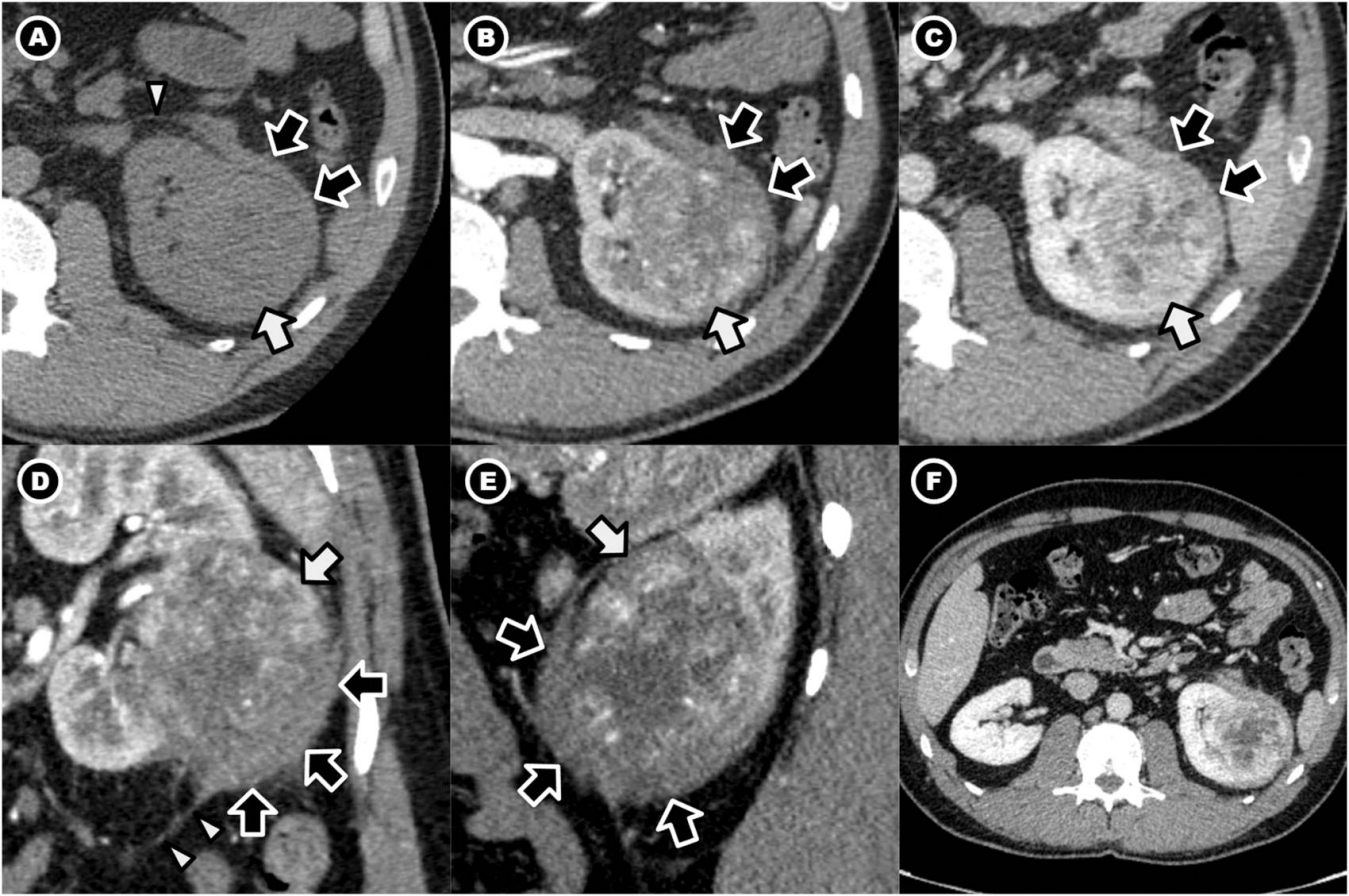
Left renal mass with adjacent perirenal soft tissue. A contrast-enhanced CT scan was performed, including non-contrast, corticomedullary (25-40 s after contrast injection), nephrographic (80-120 s after contrast injection), and excretory (5-10 min after contrast injection) phases. Axial images in the non-contrast (A), corticomedullary (B), and nephrographic (C) phases, as well as coronal (D) and sagittal (E) oblique images demonstrate a heterogeneously enhancing, predominantly endophytic mass (white arrows) in the interpolar region and inferior pole of the left kidney. An adjacent enhancing perirenal soft tissue focus (black arrows) is inseparable from the mass, infiltrates the perirenal fat, and extends beyond Gerota’s fascia (arrowheads). These findings were initially interpreted as perinephric invasion of the renal tumour, consistent with cT4 disease. The excretory phase is not shown, as it was not contributory to the renal mass evaluation. (F) Axial nephrographic phase image at the level of both kidneys demonstrates unilateral involvement confined to the left kidney and perinephric region, with no contralateral or central retroperitoneal abnormality.

During evaluation for rectal bleeding, a contrast-enhanced CT scan of the abdomen (Figure 1) was performed. Although no gastrointestinal source of bleeding was identified, imaging incidentally revealed a left renal mass with adjacent perinephric soft tissue concerning for locally advanced RCC. Based on imaging findings suggesting extension beyond Gerota’s fascia, the lesion was staged as cT4 disease. After multidisciplinary discussion, the patient proceeded to surgical management.

At open radical nephrectomy, a single large renal mass was found with dense adhesions to the surrounding perinephric tissues, diaphragm, and adjacent peritoneum. There was no clearly discernible plane of separation between the renal mass and the perinephric soft tissue. These operative findings further reinforced the preoperative impression of locally advanced disease.

Histopathologic evaluation subsequently demonstrated two distinct processes: a clear cell RCC confined to the renal parenchyma and a separate fibroinflammatory lesion involving the perinephric tissues. Postoperative ^18^F-FDG PET/CT performed six weeks after surgery demonstrated focal hypermetabolic retroperitoneal soft tissue consistent with residual IgG4-related disease and no evidence of metastatic RCC. Following confirmation of mildly elevated serum IgG4 levels, corticosteroid therapy was initiated, resulting in biochemical and imaging response on follow-up.

### Diagnostic assessment

#### Imaging-based assessment

Contrast-enhanced CT (Figure 1) demonstrated an 8.3 × 6.5 × 5.3 cm heterogeneously enhancing mass arising from the lateral midportion to lower pole of the left kidney. The lesion appeared relatively hypovascular compared with the adjacent renal cortex on the corticomedullary phase. The mass was predominantly endophytic, with no evidence of renal vein invasion, adrenal involvement, lymphadenopathy, necrosis, or calcification.

While these findings were highly suspicious for RCC, the relatively hypovascular enhancement pattern is less typical for clear cell histology and may be seen in other subtypes, including papillary RCC, chromophobe RCC, and, less commonly, urothelial carcinoma involving the renal parenchyma.

Immediately adjacent to the renal mass was an irregular perinephric soft tissue focus with indistinct margins and poor planes of separation from the tumour. This soft tissue infiltrated the perirenal fat and extended through Gerota’s fascia, features that strongly suggested perinephric tumour extension. No similar soft tissue abnormality was identified in the contralateral kidney or central retroperitoneum. Based on cross-sectional morphology alone, the perinephric component was interpreted as malignant invasion, resulting in staging as cT4 RCC.

This perinephric soft tissue component inseparable from the renal mass and extending beyond Gerota’s fascia constituted the key imaging feature leading to overstaging as cT4 disease.

#### Surgical and pathologic assessment

**Figure 2:**
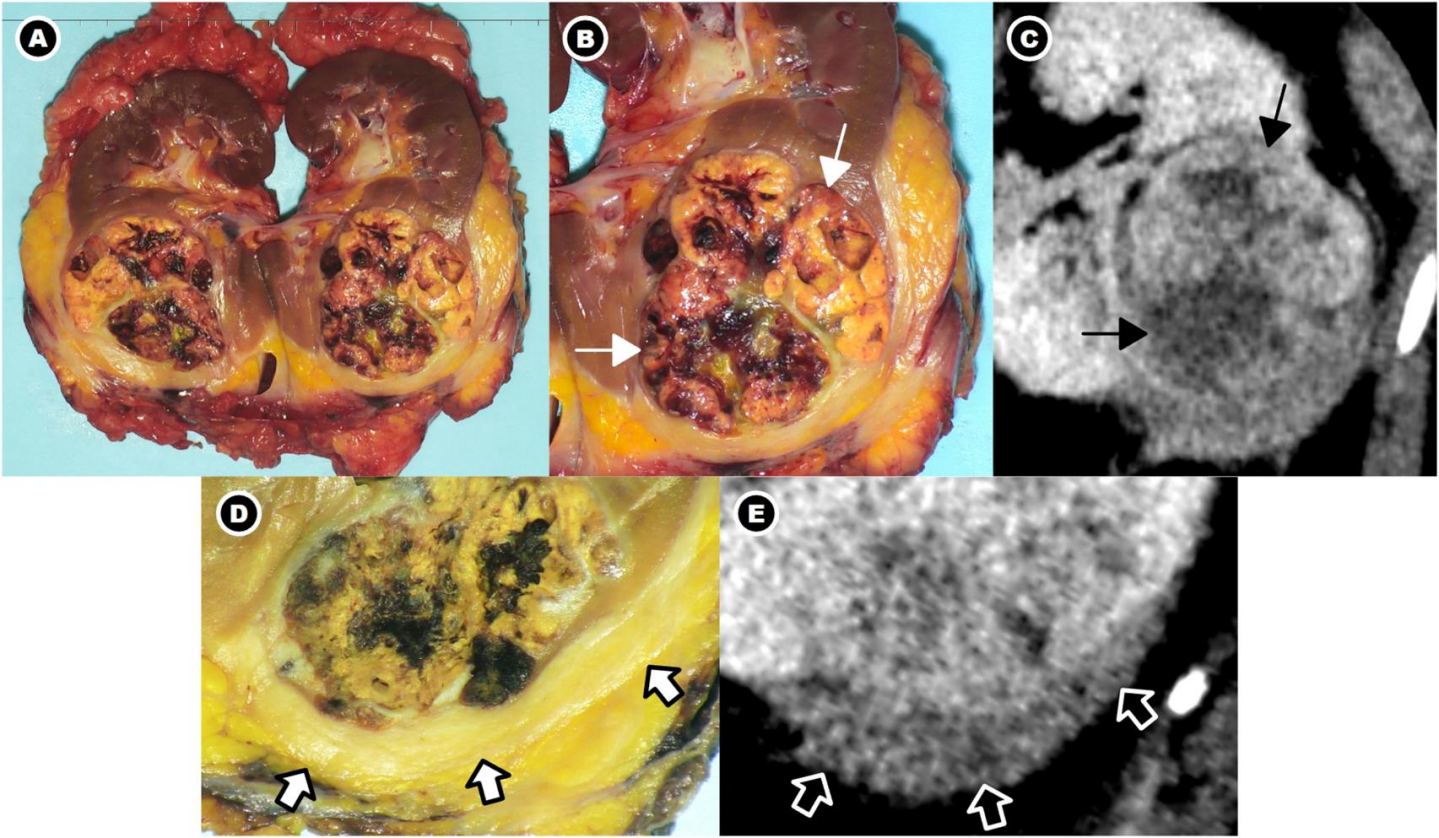
Gross pathologic findings of the left radical nephrectomy specimen with imaging correlation. (A) Gross overview of the left radical nephrectomy specimen demonstrates a renal mass in the inferior pole with an intact but thickened renal capsule and adjacent perirenal soft tissue. (B-C) Renal mass: An ovoid, unifocal mass confined to the renal parenchyma measures 6.0 × 5.5 × 4.5 cm and contains focal haemorrhagic components (white thin arrows in B), corresponding to the heterogeneously enhancing renal mass on oblique IV contrast-enhanced CT images in the nephrographic phase (black thin arrows in C). (D-E) Perirenal soft tissue: The renal capsule overlying the inferior pole mass is intact but markedly thickened, with adherent fibrous tissue (white thick arrows in D), corresponding to the perirenal soft-tissue component inseparable from the renal mass on oblique IV contrast-enhanced CT images in the nephrographic phase (black thick arrows in E).

**Figure 3:**
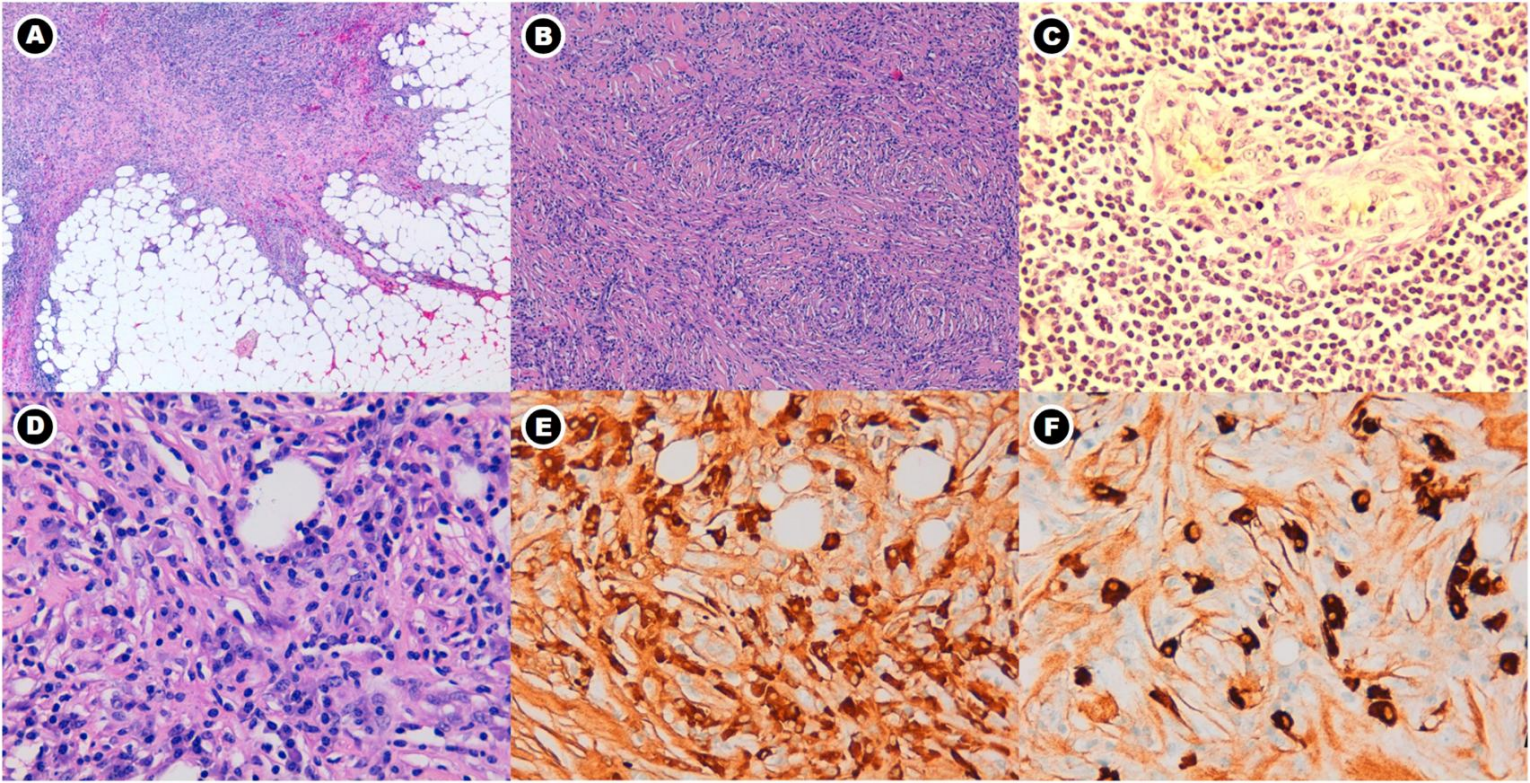
Histopathologic features of IgG4-related retroperitoneal fibrosis. (A) Haematoxylin and eosin (H&E, ×4) image demonstrates dense fibrosis with infiltrative extension into the adjacent perirenal fat. (B) H&E (×10) image shows a characteristic storiform fibrotic pattern. (C) Orcein stain (×40) highlights obliterative phlebitis within the fibroinflammatory tissue. (D) H&E (×40) image reveals a dense lymphoplasmacytic infiltrate. (E) Immunohistochemistry (×40) demonstrates numerous IgG-positive plasma cells. (F) Immunohistochemistry (×40) shows abundant IgG4-positive plasma cells (up to 200 IgG4-positive cells per high-power field; IgG4/IgG ratio 200/220), supporting the diagnosis of IgG4-related disease.

Gross examination (Figure 2) of the nephrectomy specimen demonstrated a single ovoid mass confined within the kidney, with an intact but thickened renal capsule and adjacent fibrotic tissue. Microscopic evaluation (Figure 3) confirmed clear cell RCC, grade 2, confined to the kidney (pT1b), with no perinephric fat invasion, no lymphovascular invasion, and negative surgical margins.

In contrast, the perinephric and retroperitoneal soft tissue demonstrated extensive fibroinflammatory changes characterised by storiform fibrosis, dense lymphoplasmacytic infiltrates rich in IgG4-positive plasma cells (up to 200 cells per high-power field with an IgG4+/IgG+ ratio of 91%), and focal obliterative phlebitis. These features were diagnostic of IgG4-related disease. The fibroinflammatory tissue was adherent to, but distinct from, the renal capsule, confirming that the apparent perinephric “tumour extension” represented synchronous IgG4-related RPF rather than RCC invasion.

According to the 2019 ACR/EULAR classification criteria, the immunohistochemical findings exceeded the highest diagnostic thresholds, supporting the diagnosis of IgG4-RD.^10^

#### Follow-up and outcomes

The postoperative course was complicated by a transient pneumothorax and a surgical site infection, both of which resolved with standard management.

**Figure 4:**
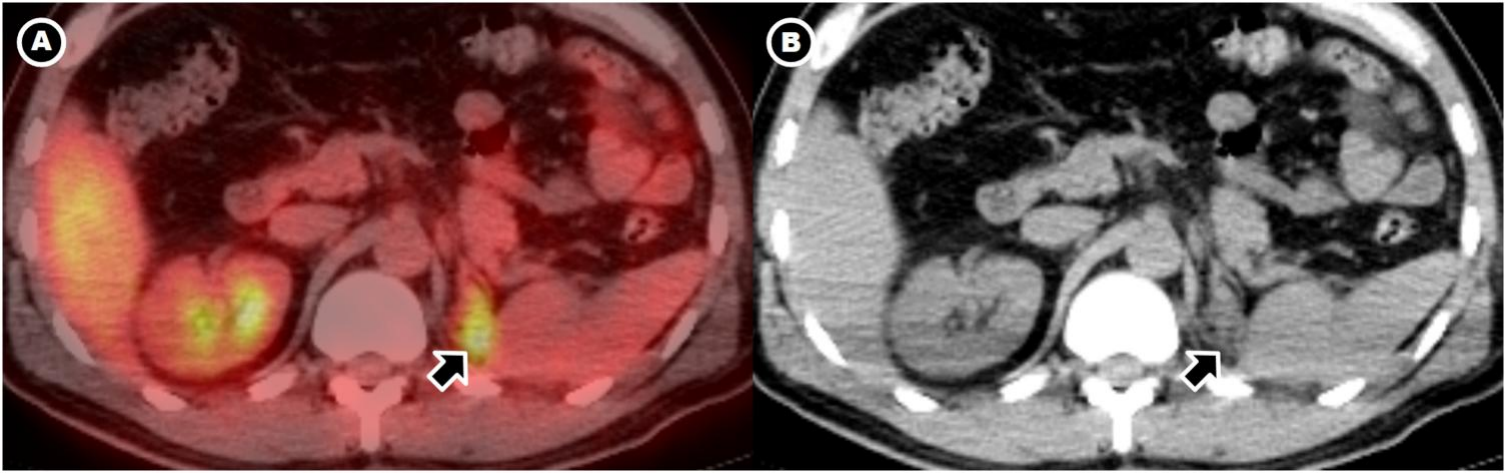
PET/CT workup demonstrating focal IgG4-related activity. Axial fused PET/CT (A) and non-contrast CT (B) through the surgical bed after IV administration of ^18^F-FDG, six weeks after left radical nephrectomy, show a moderately hypermetabolic soft-tissue focus (arrows; SUVmax 4.2) between the left diaphragmatic crus and spleen in the nephrectomy bed, consistent with active IgG4-related disease. No additional hypermetabolic foci suggestive of systemic IgG4-RD or metastatic RCC are identified, including in the contralateral kidney and central retroperitoneum.

Postoperative ^18^F-FDG PET/CT (Figure 4) demonstrated a moderately hypermetabolic retroperitoneal soft-tissue focus without additional sites of disease, supporting isolated IgG4-related RPF rather than disseminated malignancy.

**Figure 5:**
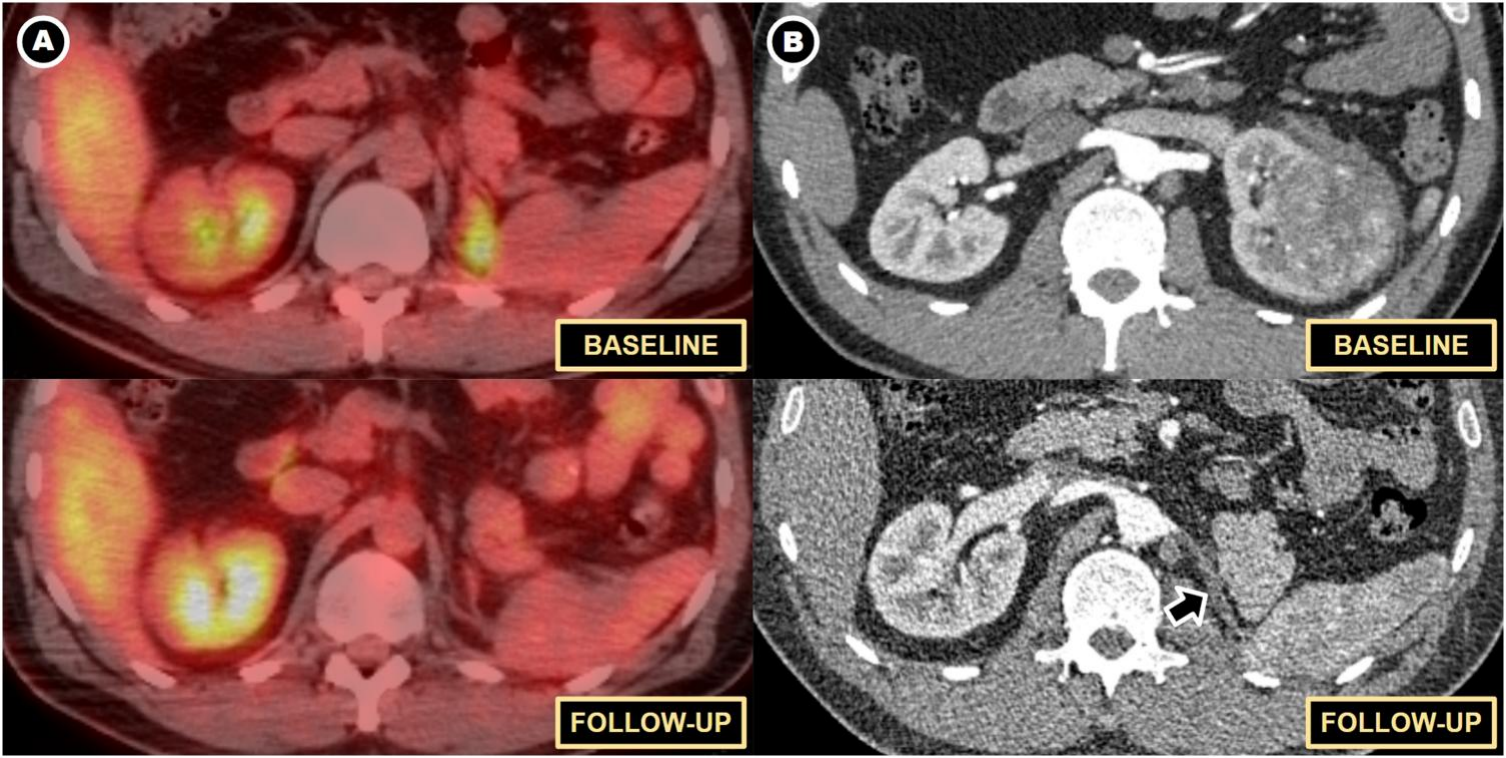
PET/CT and contrast-enhanced CT surveillance after corticosteroid therapy. (A) Axial fused PET/CT images through the left nephrectomy bed after IV administration of ^18^F-FDG, at baseline (top) and after 4 months of corticosteroid therapy (bottom), show marked metabolic regression of the previously hypermetabolic soft-tissue focus between the left diaphragmatic crus and spleen, consistent with treatment response of IgG4-related disease. No contralateral renal or retroperitoneal involvement. (B) Axial contrast-enhanced CT scan images through the left renal region at the corticomedullary phase, at preoperative baseline (top) and follow-up after 6 months of corticosteroid therapy (bottom), demonstrate no evidence of residual or recurrent renal malignancy. Only fibrotic postoperative changes (arrow) remain in the surgical bed on follow-up. No contralateral renal or retroperitoneal involvement.

Serum IgG4 was mildly elevated (94.4 mg/dL). Corticosteroid therapy was initiated one month postoperatively, resulting in progressive biochemical improvement and interval metabolic regression of the fibroinflammatory lesion on follow-up PET/CT (Figure 5) and contrast-enhanced CT. No evidence of recurrent or residual malignancy was identified. No contralateral renal or retroperitoneal involvement was identified on follow-up imaging.

## Discussion

Accurate staging of RCC is critical, as it directly influences surgical planning and prognosis. Imaging evidence of perinephric fat invasion corresponds to at least stage T3 disease, while invasion beyond Gerota’s fascia defines stage T4 disease.^3^ In the present case, CT findings strongly suggested extrarenal extension, leading to staging as cT4 RCC. However, histopathologic analysis revealed that the perinephric soft tissue represented IgG4-related RPF rather than malignant invasion, underscoring the limited specificity of imaging criteria when inflammatory disease is present.

CT plays a central role in the initial characterisation of solid renal masses by assessing enhancement, fat content, and multiphasic attenuation behaviour.^11^ Enhancement on post-contrast CT supports a solid renal neoplasm, whereas the degree and pattern of enhancement may suggest, though not definitively establish, histologic subtype. Clear cell RCC typically demonstrates strong enhancement with peak attenuation in the corticomedullary phase, whereas papillary and chromophobe RCCs are generally less avidly enhancing and more likely to peak in the nephrographic phase.^11^

In the present case, the lesion was morphologically suspicious for malignancy on CT but relatively hypovascular compared with the adjacent renal cortex on the corticomedullary phase, a pattern less typical for clear cell RCC and one that broadens the differential diagnosis to include papillary RCC, chromophobe RCC, and, in selected cases, urothelial carcinoma. This imaging appearance introduced diagnostic uncertainty not only in local staging, owing to the adjacent perinephric soft tissue, but also in subtype prediction. Nevertheless, substantial overlap among enhancement patterns of renal tumours persists, and histologic confirmation remains necessary for definitive classification.^11^

Preoperative biopsy was not pursued because the lesion was interpreted as resectable locally advanced RCC on imaging, and tissue confirmation was not expected to alter the initial management plan. Given the apparent T4 stage and absence of contraindications to surgery, radical nephrectomy was selected as the primary treatment approach. The possibility of IgG4-RD was only considered after surgical pathology demonstrated a separate fibroinflammatory process.

Other retroperitoneal processes that may involve the perinephric region, including lymphoma, Erdheim-Chester disease, and IgG4-related RPF, were not initially favoured based on imaging findings. Lymphoma typically demonstrates contiguous spread from retroperitoneal nodal disease and is commonly associated with bulky lymphadenopathy, which was absent in this case.^12^ Erdheim-Chester disease characteristically manifests as bilateral, symmetric perinephric and periaortic soft-tissue infiltration (“hairy kidney” sign) and is often accompanied by systemic involvement.^12^

IgG4-related RPF most commonly presents as plaque-like soft tissue encasing the abdominal aorta, iliac vessels, or ureters, and may extend to the perinephric space as bilateral or circumferential soft-tissue thickening rather than as a focal lesion.^1^^,^^2^^,^^12^ Focal unilateral perinephric involvement, as observed in this case, is distinctly uncommon.

In contrast, the present case demonstrated a unilateral renal mass with an adjacent focal perinephric soft-tissue component inseparable from the tumour, without contralateral or central retroperitoneal involvement. This imaging pattern strongly favoured a primary renal malignancy with perinephric extension, and alternative inflammatory or infiltrative conditions, including IgG4-RD, were not initially considered. The absence of typical imaging features of these entities contributed to the decision not to pursue further serologic evaluation or preoperative biopsy prior to surgical management.

Diagnosis of IgG4-RD requires integrated clinical, serologic, radiologic, and histopathologic assessment.^10^ Imaging reviews emphasise that IgG4-RD frequently forms mass-like lesions and may demonstrate avid FDG uptake, contributing to frequent misinterpretation as malignancy.^1^^,^^2^ IgG4-related RPF is a well-recognised malignancy mimic and has been reported to result in unnecessary surgical intervention.^4–6^ Although PET/CT is valuable for assessing disease extent and treatment response, it lacks specificity and must be interpreted in clinical context.^1^^,^^2^

This diagnostic pitfall has important management implications. Overstaging RCC may preclude nephron-sparing surgery, which is recommended for localised T1 disease when feasible, in favour of radical nephrectomy for more advanced stages.^13^ In the present case, the radiologic impression of T4 disease influenced the decision to perform radical nephrectomy.

A focused literature review (Supplementary Document 1) identified no previously reported cases of radiologically visible focal perinephric IgG4-related RPF immediately adjacent to RCC producing a direct staging mimic. The closest urologic analogue described IgG4-RD as a diagnostic trap but with a different initial misinterpretation.^5^ Although coexistence of IgG4-RD and RCC has been reported,^8^^,^^9^ adjacent perinephric involvement producing a T4 staging pitfall has not been emphasised.

An association between IgG4-RD and malignancy has been reported, though its nature remains unclear.^7^ Whether IgG4-RD represents a paraneoplastic phenomenon, a shared immune dysregulation, or a coincidental finding remains a subject of ongoing investigation. The synchronous occurrence of RCC and IgG4-related RPF in the same anatomic region in this case underscores the need for continued awareness of this potential overlap.

## Conclusion

This case highlights a rare radiologic pitfall in which focal IgG4-related retroperitoneal fibrosis mimicked perinephric extension of renal cell carcinoma, resulting in overstaging as T4 disease. When perinephric soft tissue is inseparable from a renal mass, imaging alone may be insufficient, and alternative diagnoses, including IgG4-related disease, should be considered in selected clinical contexts, particularly when accurate staging would significantly influence the feasibility of nephron-sparing management.

## Supplementary Material

uaag016_Supplementary_Data

## Learning points

1. Focal IgG4-related retroperitoneal fibrosis can rarely present as unilateral perinephric soft tissue and closely mimic tumour extension in renal cell carcinoma, leading to potential overstaging.
2. Imaging findings of perinephric soft tissue inseparable from a renal mass may appear typical of tumour invasion, and imaging alone may be insufficient for accurate staging. Histopathologic and serologic correlation are essential.
3. Consider alternative diagnoses and multidisciplinary evaluation in selected clinical contexts, particularly when accurate staging would significantly influence the feasibility of nephron-sparing management.
