# Supplementary material for: IgG4-related retroperitoneal fibrosis mimicking perinephric extension of renal cell carcinoma on CT: a case report
Source: BJR Case Rep. 2026 Apr 20;12(3):uaag016. doi: 10.1093/bjrcr/uaag016 (PMC13171608; doi:10.1093/bjrcr/uaag016)
Supplement: uaag016_Supplementary_Data [file uaag016_supplementary_data.zip › Supplementary_Document_1.docx]

# Supplementary Document 1. Literature Search for Novelty Substantiation

**1. Objective**

To determine whether any previously published cases have described a focal perinephric IgG4-related retroperitoneal fibrosis (RPF) occurring immediately adjacent to a renal cell carcinoma (RCC) and radiologically mimicking perinephric tumour extension.

**2. Databases and Sources Searched**

A comprehensive search was conducted across biomedical databases and radiology case repositories:

| **Source** | **Access Type** | **URL** |
| --- | --- | --- |
| PubMed / MEDLINE | Open access | https://pubmed.ncbi.nlm.nih.gov |
| Europe PMC | Open access | https://europepmc.org |
| Google Scholar | Open access | https://scholar.google.com |
| RSNA Case Collection | Open access | https://cases.rsna.org |
| ESR EPOS | Open access | https://epos.myesr.org |
| EURORAD | Open access | https://www.eurorad.org |
| Radiopaedia | Open access | https://radiopaedia.org |

No restrictions were placed on language, document type, publication year, or grey literature inclusion.

**3. Search Strategy**

**Date of search:** December 9, 2025

**Reviewer:** All searches were performed by one reviewer.

**Search Concepts and Terms**

| **Concept** | **Search Terms** |
| --- | --- |
| A. IgG4-related disease / retroperitoneal fibrosis | “IgG4-related disease” OR “IgG4-RD” OR “IgG4-related retroperitoneal fibrosis” OR “IgG4” |
| B. Renal cell carcinoma | “Renal cell carcinoma” OR “RCC” |
| C. Location-specific | “Perirenal” OR “peri-renal” OR “perinephric” OR “peri-nephric” |

**Combined Boolean Queries**

1. A AND B - identify IgG4-RD coexisting with RCC
2. A AND C - identify IgG4-RD involving the perirenal region
3. A AND B AND C - identify cases with both entities present specifically in the perinephric space

**4. Search Results Summary**

**Screening Table**

| **Database / Source** | **Query** | **Hits** | **Screened** | **Similar Cases** | **Notes** |
| --- | --- | --- | --- | --- | --- |
| PubMed / MEDLINE | A+B | 40 | 14 | 1 | Reviews and Case Reports |
|  | A+C | 25 | 7 | 0 | Reviews and Case Reports |
|  | A+B+C | 2 | 1 | 0 | Case Report without RCC |
| Europe PMC | A+B | 750 most relevant | 20 | 0 | Reviews, Abstracts, and Case Reports |
|  | A+C | 340 | 13 | 0 | Reviews, Abstracts, and Case Reports |
|  | A+B+C | 115 | 4 | 0 | Reviews, Abstracts, and Case Reports |
| Google Scholar | A+B | 100 most relevant | 23 | 0 | Reviews and Case Reports |
|  | A+C | 100 most relevant | 6 | 0 | Reviews and Case Reports |
|  | A+B+C | 100 most relevant | 11 | 0 | Reviews and Case Reports |
| RSNA Case Collection | IgG4 terms | 25 | 0 | 0 | Case Reports |
| ESR EPOS | IgG4 terms, with Area of Interest: Abdomen / Kidneys / Retroperitoneum | 109 | 5 | 0 | Educational Posters and Case Reports |
| EURORAD | IgG4 terms | 5 | 0 | 0 | Case Reports |
| Radiopaedia | IgG4 terms | 61 | 0 | 0 | Case Reports |

**Google Scholar and Europe PMC “A+B” Rationale**

Google Scholar retrieves thousands of heterogeneous results. Hence, only the top 100 relevance-ranked entries were reviewed because items beyond this threshold were consistently duplicated or irrelevant. The Europe PMC “A+B” also gave more than a thousand results. Hence, only the top 750 were reviewed.

**Relevance Criterion**

A relevant article required both:

1. A renal mass–perinephric lesion complex with potential to mimic malignancy, and
2. Final histopathology confirming RCC with adjacent perinephric IgG4-related disease.

**6. Article-Level Screening Summary**

| **First Author / Year** | **Title** | **Source** | **Relevance** | **Reason for Exclusion** |
| --- | --- | --- | --- | --- |
| Tricard / 2018 | [IgG4-related kidney disease: Urologist trap?]  doi: 10.1016/j.purol.2017.11.001 | PUBMED | Nearest case. pT3a ccRCC with surrounding perinephric IgG4-related retroperitoneal fibrosis, initially diagnosed as a retroperitoneal sarcoma | Pitfall was misdiagnosis as sarcoma, not RCC extension |
| Chow / 2021 | Concurrent IgG4-Related disease and clear cell renal cell carcinoma  doi: 10.1016/j.eucr.2021.101856 | PUBMED, Europe PMC, Google Scholar | ccRCC with surrounding perirenal IgG4-related disease | Surrounding perirenal IgG4-related disease not visible on imaging |
| Li / 2021 | Case Report of Patient with IgG4-Related Disease and Renal Cell Carcinoma  doi: 10.21203/rs.3.rs-169125/v1 | Europe PMC, Google Scholar | ccRCC with surrounding perirenal IgG4-related disease | Surrounding perirenal IgG4-related disease not visible on imaging and within renal parenchyma |
| Watanabe / 2014 | Distinct cytokine mRNA expression pattern in immunoglobulin G4-related kidney disease associated with renal cell carcinoma  doi: 10.1093/ckj/sfu024 | PUBMED, Europe PMC, Google Scholar | ccRCC with renal parenchymal IgG4 infiltration | IgG4-related disease of the kidney not visible on imaging and within kidney |
| Khan / 2014 | Renal cell carcinoma mimicking IgG4-related pseudotumor in autoimmune pancreatitis  doi: 10.6092/1590-8577/2808 | PUBMED | IgG4 autoimmune pancreatitis with associated RCC | IgG4-RD and RCC not in the same location |
| Mathew / 2025 | Rare case of IgG4-related retroperitoneal fibrosis mimicking renal cell carcinoma: A diagnostic challenge  doi: 10.4103/ijpm.ijpm_568_24 | PUBMED, Europe PMC, Google Scholar | IgG4-RD mimicking a renal malignancy | Without RCC |
| Yamamoto / 2019 | [A Case of Bilateral Renal Tumors, Renal Cell Carcinoma and IgG4- Related Retroperitoneal Fibrosis]  doi: 10.14989/ActaUrolJap_65_10_407 | PUBMED, Europe PMC, Google Scholar | IgG4-RD in the right kidney and RCC in the left kidney | IgG4-RD and RCC not in the same location |
| Cai / 2016 | IgG4-related inflammatory pseudotumor of the kidney mimicking renal cell carcinoma: A case report  doi: 10.3892/ol.2016.4408 | PUBMED, Europe PMC, Google Scholar | IgG4-RD mimicking a renal malignancy | Without RCC |
| Watanabe / 2019 | A case of immunoglobulin G4-related inflammatory pseudotumor mimicking renal cell carcinoma  doi: 10.1007/s00261-018-01885-1 | PUBMED, Europe PMC | IgG4-RD mimicking a renal malignancy | Without RCC |
| Nofuji / 2013 | [A case of IgG4-related kidney disease mimicking a renal cell carcinoma] | PUBMED, Google Scholar | IgG4-RD mimicking a renal malignancy | Without RCC |
| Wu / 2020 | Renal pseudotumor: A new challenge in the diagnosis of immunoglobulin G4-related disease  doi: 10.4103/jcrt.JCRT_697_20 | PUBMED, Europe PMC | IgG4-RD mimicking a renal malignancy | Without RCC |
| Inoue / 2025 | Immunoglobulin G4-related Autoimmune Pancreatitis and Hypopituitarism Following Immune Checkpoint Inhibitor Therapy  doi: 10.2169/internalmedicine.3591-24 | PUBMED, Europe PMC, Google Scholar | IgG4-related AIP after nephrectomy for RCC | IgG4-RD and RCC not in the same location |
| Oae / 2011 | [IgG4-related tubulointerstitial nephritis presented with multiple renal nodular lesions] | PUBMED | Bilateral renal nodules, diagnosed as IgG4-related TIN, after a history of RCC and AIP | IgG4-RD within the renal parenchyma, not the perinephric region |
| Miura / 2008 | An autopsy case of autoimmune pancreatitis after a 6-year history of steroid therapy accompanied by malignant dissemination of unknown origin  doi: 10.1097/MEG.0b013e3282f555ad | PUBMED | IgG4-related AIP with metastatic RCC | IgG4-RD and RCC not in the same location |
| Wang / 2023 | Perirenal IgG4-related disease  doi: 10.1016/j.kint.2022.10.011 | PUBMED, Europe PMC, Google Scholar | Perinephric IgG4-related RPF | Without RCC |
| Matsubara / 2023 | IgG4-related Disease with Localized Perirenal Soft-tissue Thickening  doi: 10.2169/internalmedicine.9785-22 | PUBMED, Europe PMC | Perinephric IgG4-related RPF | Without RCC |
| Cho / 2018 | Perirenal capsule and scrotal involvement in immunoglobulin G4-related kidney disease: case-based review  doi: 10.1007/s00296-018-4089-y | PUBMED, Google Scholar | Perinephric capsule IgG4-RD | Without RCC |
| He / 2022 | Immunoglobulin G4-related kidney disease involving the renal pelvis and perirenal fat: A case report  doi: 10.12998/wjcc.v10.i8.2510 | PUBMED, Europe PMC, Google Scholar | Perinephric IgG4-related RPF | Without RCC |
| Zhou / 2025 | A case of IgG4-related disease misdiagnosed as perirenal abscess: Case report and literature review  doi: 10.1097/MD.0000000000045898 | PUBMED, Europe PMC, Google Scholar | Perinephric RPF mimicking a renal abscess. | Without RCC |
| Endo / 2021 | Rapidly Progressive Kidney Failure Associated with Perirenal Capsular Lesion Due to IgG4-Related Disease  doi: 10.2169/internalmedicine.6232-20 | PUBMED, Google Scholar | Perinephric IgG4-related RPF | Without RCC |
| Kobayashi / 2024 | Immunoglobulin G4-related disease manifesting as peripheral neuropathy: A rare clinical symptom due to rare autoimmune disease  doi: 10.25259/SNI_157_2024 | PUBMED | Perinephric IgG4-related RPF | Without RCC |
| Shrivastava / 2023 | Primary retroperitoneal fibrosis presenting as a renal mass.  doi: 10.4103/iju.iju_391_22 | PUBMED, Europe PMC, Google Scholar | Perinephric RPF mimicking a renal mass. | Without RCC |
| Tanaka / 2025 | Immunoglobulin G4-Related Inflammatory Pseudotumor With Cystic Features Mimicking Renal Cancer.  doi: 10.1002/iju5.70074 | Europe PMC, Google Scholar | IgG4-RD mimicking a renal malignancy | Without RCC |
| Minezaki / 2025 | An Atypical Cystic Renal Mass in a Patient with IgG4-Related Kidney Disease.  doi: 10.2169/internalmedicine.4691-24 | Europe PMC | IgG4-RD mimicking a renal malignancy | Without RCC |
| Althammer / 2024 | Renal pseudotumor mimicking renal cell carcinoma in an elderly patient with ovarian carcinoma: Case report and literature review.  doi: 10.1016/j.radcr.2024.11.063 | Europe PMC | IgG4-RD mimicking a renal malignancy | Without RCC |
| Tawhari / 2022 | IgG4-Related Kidney Disease Associated With End-Stage Kidney Disease, Renal Pseudotumor, and Renal Vein Thrombosis.  doi: 10.7759/cureus.22837 | Europe PMC, Google Scholar | IgG4-RD mimicking a renal malignancy | Without RCC |
| Ng / 2021 | IgG4-related disease: an atypical presentation of steroid-responsive renal mass.  doi: 10.1136/bcr-2020-240611 | Europe PMC, Google Scholar | IgG4-RD mimicking a renal malignancy | Without RCC |
| Liu / 2021 | Inflammatory pseudotumor of Castleman disease and IgG4-related disease masquerading as kidney malignancy.  doi: 10.1186/s13000-021-01134-y | Europe PMC, Google Scholar | IgG4-RD mimicking a renal malignancy | Without RCC |
| Samji / 2020 | A Case of an IgG4-Related Disease Mimicking Malignancy and Resolving With Steroids.  doi: 10.7759/cureus.9476 | Europe PMC, Google Scholar | IgG4-RD mimicking a renal malignancy | Without RCC |
| Wang/ 2014 | IgG4-related systemic disease mimicking renal pelvic cancer: a rare case.  doi: 10.1186/1477-7819-12-395 | Europe PMC, Google Scholar | IgG4-RD mimicking a renal pelvic cancer | Without RCC |
| Xu / 2017 | Clinicopathological analysis of renal inflammatory pseudotumors presenting as the unilateral solitary masses. | Europe PMC, Google Scholar | IgG4-RD mimicking a renal malignancy | Without RCC |
| Alkhasawneh / 2012 | IgG4 Inflammatory Pseudotumor of the Kidney.  doi: 10.1155/2012/919087 | Europe PMC | IgG4-RD mimicking a renal malignancy | Without RCC |
| Kim / 2013 | Immunoglobulin g4-related systemic sclerosing disease: a case involving the ureter and kidney.  doi: 10.4111/kju.2013.54.3.209 | Europe PMC | IgG4-RD mimicking a mass involving the ureter and kidney | Without RCC |
| Chen / 2018 | Perirenal soft tissue infiltration from immunoglobulin G4–related disease  doi: 10.1503/cmaj.180264 | Europe PMC | IgG4-related RPF surrounding both kidneys | Without RCC |
| Mehta / 2012 | Immunoglobulin G4-related sclerosing disease presenting as a rare cause of renal pelvic mass mimicking malignancy.  doi: 10.2484/rcr.v7i4.755 | Europe PMC | IgG4-related RPF mimicking a malignant renal pelvic mass | Without RCC |
| Yoshino / 2013 | A Case of IgG4-Related Retroperitoneal Fibrosis Mimicking Renal Pelvic Cancer  doi: 10.1159/000341703 | Google Scholar | IgG4-related RPF mimicking a malignant renal pelvic mass | Without RCC |
| Zhou / 2021 | IgG4-Related Disease as Mimicker of Malignancy  doi: 10.1007/s42399-021-00957-6 | Google Scholar | IgG4-RD mimicking a malignancy | Without RCC |
| Park / 2016 | IgG4-related inflammatory pseudotumor of the renal pelvis involving renal parenchyma, mimicking malignancy  doi: 10.1186/s13000-016-0460-z | Google Scholar | IgG4-related RPF mimicking a malignant renal pelvic mass | Without RCC |
| Nuthalapati / 2025 | IgG4-Related Disease in Urological Practice: A Case Series of Mistaken Malignancies  doi: 10.1007/s13193-025-02233-8 | Google Scholar | IgG4-RD mimicking a malignancy | Without RCC |
| Thia / 2023 | An uncommon mimicker of renal malignancy: IgG4-related disease  doi: 10.1186/s12894-023-01304-8 | Google Scholar | IgG4-related RPF mimicking a malignant renal pelvic mass | Without RCC |
| Lee / 2013 | IgG4-related sclerosing disease in the kidney – a case report  doi: 10.1097/01.PAT.0000426905.09167.93 | Google Scholar | IgG4-related RPF mimicking a malignant renal pelvic mass | Without RCC |
| Nakada / 2022 | A case of IgG4-related retroperitoneal fibrosis diagnosed by tissue biopsy  doi: 10.5387/fmedj.72.2_65 | Google Scholar | IgG4-related disease mimicking a malignant renal pelvic mass | Without RCC |
| Olivaira / 2021 | IgG4-related disease with renal and orbital involvement: a clinical case | Google Scholar | IgG4-related disease mimicking a renal mass | Without RCC |
| Yu / 2021 | A 4-Year-old Boy With Right Renal Space-occupying Lesion Diagnosed With Inflammatory Pseudotumor  doi: 10.1016/j.urology.2020.11.003 | Google Scholar | IgG4-related disease mimicking a malignant renal mass | Without RCC |
| Mukkamala / 2018 | Open-access Inflammatory pseudotumor of kidney: a challenging diagnostic entity  doi: 10.1590/S1677-5538.IBJU.2017.0063 | Google Scholar | IgG4-related disease mimicking a malignancy | Without RCC |
| Han / 2023 | The MRI features of renal inflammatory pseudotumor: A case report and literature review  doi: 10.1097/MD.0000000000033287 | Google Scholar | IgG4-related disease mimicking a malignancy | Without RCC |
| Brunie / 2018 | Renal Pseudotumors: Features that help differentiate them from a real neoplasm.  doi: 10.1594/ecr2018/C-2488 | ESR EPOS | IgG4-related disease mimicking a malignancy | Without RCC |
| Patel / 2020 | Retroperitoneal fibrosis and mimics: a multimodality pictorial review  doi: 10.26044/ecr2020/C-10316 | ESR EPOS | IgG4-related disease mimicking a malignancy | Without RCC |
| Rato / 2025 | The great mimickers of abdominal malignancy  doi: 10.26044/ecr2025/C-19953 | ESR EPOS | IgG4-related disease mimicking a malignancy | Without RCC |
| Jyani / 2022 | IgG4-related disease: A great mimicker  doi: 10.26044/ecr2022/C-10161 | ESR EPOS | IgG4-related disease mimicking a malignancy | Without RCC |
| Kim / 2013 | CT and MR Image Features of Retroperitoneal Fibrosis-Mimicking Soft Tissue Diseases involving Urinary System with Literature Review  doi: 10.1594/ecr2013/C-1270 | ESR EPOS | IgG4-related disease mimicking a malignancy | Without RCC |

Total screened across all databases: 51

Cases involving IgG4-RD adjacent to RCC on imaging or final histopathology: 1

**Interpretation of the Single Similar Case (Tricard 2018)**

Only one published case (Tricard 2018) described RCC with adjacent IgG4-related fibrosis. However:

- The diagnostic pitfall involved misinterpreting the combined mass as a retroperitoneal sarcoma extending into the kidney, not as RCC extension into the perinephric fat.
- RCC was stage pT3a, whereas in our case, a small pT1b RCC was misinterpreted as T4, representing a more significant staging discrepancy.

**Reasons Other Cases Were Excluded**

Most screened reports featured:

- IgG4-RD without RCC
- IgG4-RD confined to the renal parenchyma, even when adjacent to an RCC
- IgG4-RD and RCC in different anatomic locations
- IgG4-RD detected only microscopically, invisible on imaging

No prior publication described a radiologically visible focal perinephric IgG4-RPF abutting an RCC and simulating extrarenal tumour extension.

**7. Conclusion**

Across all databases, no prior cases described a focal IgG4-related retroperitoneal fibrosis lesion immediately adjacent to a renal cell carcinoma and radiologically mimicking perinephric tumour extension (T4 disease).

Thus, this appears to be the first reported case of radiologically visible perinephric IgG4-related fibrosis directly adjacent to a renal cell carcinoma and mimicking its extrarenal extension, supporting the novelty of the case as of December 9, 2025.

**8. Search Limitations**

- Some queries were limited (Google Scholar screens up to 100 and Europe PMC A+B screens up to 750) because entries beyond this threshold were consistently duplicative or irrelevant.
- Searches were performed by a single reviewer due to the narrow scope of the query and the descriptive nature of novelty substantiation. However, this may introduce a minor risk of missed studies despite the broad search strategy.
- Some older case reports may exist in non-indexed regional journals not captured by the searched databases, though this is unlikely to affect the conclusion.
