# Supplementary material for: IgG4-related retroperitoneal fibrosis mimicking perinephric extension of renal cell carcinoma on CT: a case report
Source: BJR Case Rep. 2026 Apr 20;12(3):uaag016. doi: 10.1093/bjrcr/uaag016 (PMC13171608; doi:10.1093/bjrcr/uaag016)
Supplement: uaag016_Supplementary_Data [file uaag016_supplementary_data.zip › 27-Apr-2026_012942_Supplementary_Document_1.pdf]

Supplementary Document 1. Literature Search for Novelty Substantiation

1. Objective

To determine whether any previously published cases have described a focal perinephric IgG4-related retroperitoneal fibrosis (RPF) occurring immediately adjacent to a renal cell carcinoma (RCC) and radiologically mimicking perinephric tumour extension.

2. Databases and Sources Searched

A comprehensive search was conducted across biomedical databases and radiology case repositories:

Source	Access Type	URL
PubMed / MEDLINE	Open access	https://pubmed.ncbi.nlm.nih.gov
Europe PMC	Open access	https://europepmc.org
Google Scholar	Open access	https://scholar.google.com
RSNA Case Collection	Open access	https://cases.rsna.org
ESR EPOS	Open access	https://epos.myesr.org
EURORAD	Open access	https://www.eurorad.org
Radiopaedia	Open access	https://radiopaedia.org

No restrictions were placed on language, document type, publication year, or grey literature inclusion.

3. Search Strategy

Date of search: December 9, 2025

Reviewer: All searches were performed by one reviewer.

Search Concepts and Terms

Concept	Search Terms
A. IgG4-related disease / retroperitoneal fibrosis	"IgG4-related disease" OR "IgG4-RD" OR "IgG4-related retroperitoneal fibrosis" OR "IgG4"
B. Renal cell carcinoma	"Renal cell carcinoma" OR "RCC"
C. Location-specific	"Perirenal" OR "peri-renal" OR "perinephric" OR "peri-nephric"

Combined Boolean Queries

1. A AND B - identify IgG4-RD coexisting with RCC
2. A AND C - identify IgG4-RD involving the perirenal region
3. A AND B AND C - identify cases with both entities present specifically in the perinephric space

4. Search Results Summary

Screening Table

Database / Source	Query	Hits	Screened	Similar Cases	Notes
PubMed / MEDLINE	A+B	40	14	1	Reviews and Case Reports
	A+C	25	7	0	Reviews and Case Reports
	A+B+C	2	1	0	Case Report without RCC
Europe PMC	A+B	750 most relevant	20	0	Reviews, Abstracts, and Case Reports
	A+C	340	13	0	Reviews, Abstracts, and Case Reports
	A+B+C	115	4	0	Reviews, Abstracts, and Case Reports
Google Scholar	A+B	100 most relevant	23	0	Reviews and Case Reports
	A+C	100 most relevant	6	0	Reviews and Case Reports
	A+B+C	100 most relevant	11	0	Reviews and Case Reports
RSNA Case Collection	IgG4 terms	25	0	0	Case Reports
ESR EPOS	IgG4 terms, with Area of Interest: Abdomen / Kidneys / Retroperitoneum	109	5	0	Educational Posters and Case Reports
EURORAD	IgG4 terms	5	0	0	Case Reports
Radiopaedia	IgG4 terms	61	0	0	Case Reports

Google Scholar and Europe PMC "A+B" Rationale

Google Scholar retrieves thousands of heterogeneous results. Hence, only the top 100 relevance-ranked entries were reviewed because items beyond this threshold were consistently duplicated or irrelevant. The Europe PMC "A+B" also gave more than a thousand results. Hence, only the top 750 were reviewed.

Relevance Criterion

A relevant article required both:

1. A renal mass–perinephric lesion complex with potential to mimic malignancy, and
2. Final histopathology confirming RCC with adjacent perinephric IgG4-related disease.

6. Article-Level Screening Summary

First Author / Year	Title	Source	Relevance	Reason for Exclusion
Tricard / 2018	[IgG4-related kidney disease: Urologist trap?]<br>doi: 10.1016/j.purol.2017.11.001	PUBMED	Nearest case. pT3a ccRCC with surrounding perinephric IgG4-related retroperitoneal fibrosis, initially diagnosed as a retroperitoneal sarcoma	Pitfall was misdiagnosis as sarcoma, not RCC extension
Chow / 2021	Concurrent IgG4-Related disease and clear cell renal cell carcinoma<br>doi: 10.1016/j.eucr.2021.101856	PUBMED, Europe PMC, Google Scholar	ccRCC with surrounding perirenal IgG4-related disease	Surrounding perirenal IgG4-related disease not visible on imaging
Li / 2021	Case Report of Patient with IgG4-Related Disease and Renal Cell Carcinoma<br>doi: 10.21203/rs.3.rs-169125/v1	Europe PMC, Google Scholar	ccRCC with surrounding perirenal IgG4-related disease	Surrounding perirenal IgG4-related disease not visible on imaging and within renal parenchyma
Watanabe / 2014	Distinct cytokine mRNA expression pattern in immunoglobulin G4-related kidney disease associated with renal cell carcinoma<br>doi: 10.1093/ckj/sfu024	PUBMED, Europe PMC, Google Scholar	ccRCC with renal parenchymal IgG4 infiltration	IgG4-related disease of the kidney not visible on imaging and within kidney
Khan / 2014	Renal cell carcinoma mimicking IgG4-related pseudotumor in autoimmune pancreatitis<br>doi: 10.6092/1590-8577/2808	PUBMED	IgG4 autoimmune pancreatitis with associated RCC	IgG4-RD and RCC not in the same location
Mathew / 2025	Rare case of IgG4-related retroperitoneal fibrosis mimicking renal cell carcinoma: A diagnostic challenge<br>doi: 10.4103/ijpm.ijpm_568_24	PUBMED, Europe PMC, Google Scholar	IgG4-RD mimicking a renal malignancy	Without RCC
Yamamoto / 2019	[A Case of Bilateral Renal Tumors, Renal Cell Carcinoma and IgG4- Related Retroperitoneal Fibrosis]<br>doi: 10.14989/ActaUrolJap_65_10_407	PUBMED, Europe PMC, Google Scholar	IgG4-RD in the right kidney and RCC in the left kidney	IgG4-RD and RCC not in the same location
Cai / 2016	IgG4-related inflammatory pseudotumor of the kidney mimicking renal cell carcinoma: A case report<br>doi: 10.3892/ol.2016.4408	PUBMED, Europe PMC, Google Scholar	IgG4-RD mimicking a renal malignancy	Without RCC
Watanabe / 2019	A case of immunoglobulin G4-related inflammatory pseudotumor mimicking renal cell carcinoma<br>doi: 10.1007/s00261-018-01885-1	PUBMED, Europe PMC	IgG4-RD mimicking a renal malignancy	Without RCC
Nofuji / 2013	[A case of IgG4-related kidney disease mimicking a renal cell carcinoma]	PUBMED, Google Scholar	IgG4-RD mimicking a renal malignancy	Without RCC
Wu / 2020	Renal pseudotumor: A new challenge in the diagnosis of immunoglobulin G4-related disease<br>doi: 10.4103/jcrt.JCRT_697_20	PUBMED, Europe PMC	IgG4-RD mimicking a renal malignancy	Without RCC
Inoue / 2025	Immunoglobulin G4-related Autoimmune Pancreatitis and Hypopituitarism Following Immune Checkpoint Inhibitor Therapy<br>doi: 10.2169/internalmedicine.3591-24	PUBMED, Europe PMC, Google Scholar	IgG4-related AIP after nephrectomy for RCC	IgG4-RD and RCC not in the same location
Oae / 2011	[IgG4-related tubulointerstitial nephritis presented with multiple renal nodular lesions]	PUBMED	Bilateral renal nodules, diagnosed as IgG4-related TIN,	IgG4-RD within the renal parenchyma, not the perinephric region

			after a history of RCC and AIP	
Miura / 2008	An autopsy case of autoimmune pancreatitis after a 6-year history of steroid therapy accompanied by malignant dissemination of unknown origin<br>doi: 10.1097/MEG.0b013e3282f555ad	PUBMED	IgG4-related AIP with metastatic RCC	IgG4-RD and RCC not in the same location
Wang / 2023	Perirenal IgG4-related disease<br>doi: 10.1016/j.kint.2022.10.011	PUBMED, Europe PMC, Google Scholar	Perinephric IgG4-related RPF	Without RCC
Matsubara / 2023	IgG4-related Disease with Localized Perirenal Soft-tissue Thickening<br>doi: 10.2169/internalmedicine.9785-22	PUBMED, Europe PMC	Perinephric IgG4-related RPF	Without RCC
Cho / 2018	Perirenal capsule and scrotal involvement in immunoglobulin G4-related kidney disease: case-based review<br>doi: 10.1007/s00296-018-4089-y	PUBMED, Google Scholar	Perinephric capsule IgG4-RD	Without RCC
He / 2022	Immunoglobulin G4-related kidney disease involving the renal pelvis and perirenal fat: A case report<br>doi: 10.12998/wjcc.v10.i8.2510	PUBMED, Europe PMC, Google Scholar	Perinephric IgG4-related RPF	Without RCC
Zhou / 2025	A case of IgG4-related disease misdiagnosed as perirenal abscess: Case report and literature review<br>doi: 10.1097/MD.0000000000045898	PUBMED, Europe PMC, Google Scholar	Perinephric RPF mimicking a renal abscess.	Without RCC
Endo / 2021	Rapidly Progressive Kidney Failure Associated with Perirenal Capsular Lesion Due to IgG4-Related Disease<br>doi: 10.2169/internalmedicine.6232-20	PUBMED, Google Scholar	Perinephric IgG4-related RPF	Without RCC
Kobayashi / 2024	Immunoglobulin G4-related disease manifesting as peripheral neuropathy: A rare clinical symptom due to rare autoimmune disease<br>doi: 10.25259/SNI_157_2024	PUBMED	Perinephric IgG4-related RPF	Without RCC
Shrivastava / 2023	Primary retroperitoneal fibrosis presenting as a renal mass.<br>doi: 10.4103/iju.iju_391_22	PUBMED, Europe PMC, Google Scholar	Perinephric RPF mimicking a renal mass.	Without RCC
Tanaka / 2025	Immunoglobulin G4-Related Inflammatory Pseudotumor With Cystic Features Mimicking Renal Cancer.<br>doi: 10.1002/iju5.70074	Europe PMC, Google Scholar	IgG4-RD mimicking a renal malignancy	Without RCC
Minezaki / 2025	An Atypical Cystic Renal Mass in a Patient with IgG4-Related Kidney Disease.<br>doi: 10.2169/internalmedicine.4691-24	Europe PMC	IgG4-RD mimicking a renal malignancy	Without RCC
Althammer / 2024	Renal pseudotumor mimicking renal cell carcinoma in an elderly patient with ovarian carcinoma: Case report and literature review.<br>doi: 10.1016/j.radcr.2024.11.063	Europe PMC	IgG4-RD mimicking a renal malignancy	Without RCC
Tawhari / 2022	IgG4-Related Kidney Disease Associated With End-Stage Kidney Disease, Renal Pseudotumor, and Renal Vein Thrombosis.<br>doi: 10.7759/cureus.22837	Europe PMC, Google Scholar	IgG4-RD mimicking a renal malignancy	Without RCC
Ng / 2021	IgG4-related disease: an atypical presentation of steroid-responsive renal mass.<br>doi: 10.1136/bcr-2020-240611	Europe PMC, Google Scholar	IgG4-RD mimicking a renal malignancy	Without RCC
Liu / 2021	Inflammatory pseudotumor of Castleman disease and IgG4-related disease masquerading as kidney malignancy.<br>doi: 10.1186/s13000-021-01134-y	Europe PMC, Google Scholar	IgG4-RD mimicking a renal malignancy	Without RCC
Samji / 2020	A Case of an IgG4-Related Disease Mimicking Malignancy and Resolving With Steroids.	Europe PMC, Google Scholar	IgG4-RD mimicking a renal malignancy	Without RCC

	doi: 10.7759/cureus.9476			
Wang/ 2014	IgG4-related systemic disease mimicking renal pelvic cancer: a rare case.<br>doi: 10.1186/1477-7819-12-395	Europe PMC, Google Scholar	IgG4-RD mimicking a renal pelvic cancer	Without RCC
Xu / 2017	Clinicopathological analysis of renal inflammatory pseudotumors presenting as the unilateral solitary masses.	Europe PMC, Google Scholar	IgG4-RD mimicking a renal malignancy	Without RCC
Alkhasawneh / 2012	IgG4 Inflammatory Pseudotumor of the Kidney.<br>doi: 10.1155/2012/919087	Europe PMC	IgG4-RD mimicking a renal malignancy	Without RCC
Kim / 2013	Immunoglobulin g4-related systemic sclerosing disease: a case involving the ureter and kidney.<br>doi: 10.4111/kju.2013.54.3.209	Europe PMC	IgG4-RD mimicking a mass involving the ureter and kidney	Without RCC
Chen / 2018	Perirenal soft tissue infiltration from immunoglobulin G4–related disease<br>doi: 10.1503/cmaj.180264	Europe PMC	IgG4-related RPF surrounding both kidneys	Without RCC
Mehta / 2012	Immunoglobulin G4-related sclerosing disease presenting as a rare cause of renal pelvic mass mimicking malignancy.<br>doi: 10.2484/rcr.v7i4.755	Europe PMC	IgG4-related RPF mimicking a malignant renal pelvic mass	Without RCC
Yoshino / 2013	A Case of IgG4-Related Retroperitoneal Fibrosis Mimicking Renal Pelvic Cancer<br>doi: 10.1159/000341703	Google Scholar	IgG4-related RPF mimicking a malignant renal pelvic mass	Without RCC
Zhou / 2021	IgG4-Related Disease as Mimicker of Malignancy<br>doi: 10.1007/s42399-021-00957-6	Google Scholar	IgG4-RD mimicking a malignancy	Without RCC
Park / 2016	IgG4-related inflammatory pseudotumor of the renal pelvis involving renal parenchyma, mimicking malignancy<br>doi: 10.1186/s13000-016-0460-z	Google Scholar	IgG4-related RPF mimicking a malignant renal pelvic mass	Without RCC
Nuthalapati / 2025	IgG4-Related Disease in Urological Practice: A Case Series of Mistaken Malignancies<br>doi: 10.1007/s13193-025-02233-8	Google Scholar	IgG4-RD mimicking a malignancy	Without RCC
Thia / 2023	An uncommon mimicker of renal malignancy: IgG4-related disease<br>doi: 10.1186/s12894-023-01304-8	Google Scholar	IgG4-related RPF mimicking a malignant renal pelvic mass	Without RCC
Lee / 2013	IgG4-related sclerosing disease in the kidney – a case report<br>doi: 10.1097/01.PAT.0000426905.09167.93	Google Scholar	IgG4-related RPF mimicking a malignant renal pelvic mass	Without RCC
Nakada / 2022	A case of IgG4-related retroperitoneal fibrosis diagnosed by tissue biopsy<br>doi: 10.5387/fmedj.72.2_65	Google Scholar	IgG4-related disease mimicking a malignant renal pelvic mass	Without RCC
Olivaira / 2021	IgG4-related disease with renal and orbital involvement: a clinical case	Google Scholar	IgG4-related disease mimicking a renal mass	Without RCC
Yu / 2021	A 4-Year-old Boy With Right Renal Space-occupying Lesion Diagnosed With Inflammatory Pseudotumor<br>doi: 10.1016/j.urology.2020.11.003	Google Scholar	IgG4-related disease mimicking a malignant renal mass	Without RCC
Mukkamala / 2018	Open-access Inflammatory pseudotumor of kidney: a challenging diagnostic entity<br>doi: 10.1590/S1677-5538.IBJU.2017.0063	Google Scholar	IgG4-related disease mimicking a malignancy	Without RCC

Han / 2023	The MRI features of renal inflammatory pseudotumor: A case report and literature review<br>doi: 10.1097/MD.0000000000033287	Google Scholar	IgG4-related disease mimicking a malignancy	Without RCC
Brunie / 2018	Renal Pseudotumors: Features that help differentiate them from a real neoplasm.<br>doi: 10.1594/ecr2018/C-2488	ESR EPOS	IgG4-related disease mimicking a malignancy	Without RCC
Patel / 2020	Retroperitoneal fibrosis and mimics: a multimodality pictorial review<br>doi: 10.26044/ecr2020/C-10316	ESR EPOS	IgG4-related disease mimicking a malignancy	Without RCC
Rato / 2025	The great mimickers of abdominal malignancy<br>doi: 10.26044/ecr2025/C-19953	ESR EPOS	IgG4-related disease mimicking a malignancy	Without RCC
Jyani / 2022	IgG4-related disease: A great mimicker<br>doi: 10.26044/ecr2022/C-10161	ESR EPOS	IgG4-related disease mimicking a malignancy	Without RCC
Kim / 2013	CT and MR Image Features of Retroperitoneal Fibrosis-Mimicking Soft Tissue Diseases involving Urinary System with Literature Review<br>doi: 10.1594/ecr2013/C-1270	ESR EPOS	IgG4-related disease mimicking a malignancy	Without RCC

Total screened across all databases: 51
Cases involving IgG4-RD adjacent to RCC on imaging or final histopathology: 1

Interpretation of the Single Similar Case (Tricard 2018)
Only one published case (Tricard 2018) described RCC with adjacent IgG4-related fibrosis. However:
- The diagnostic pitfall involved misinterpreting the combined mass as a retroperitoneal sarcoma extending into the kidney, not as RCC extension into the perinephric fat.
- RCC was stage pT3a, whereas in our case, a small pT1b RCC was misinterpreted as T4, representing a more significant staging discrepancy.

Reasons Other Cases Were Excluded
Most screened reports featured:
- IgG4-RD without RCC
- IgG4-RD confined to the renal parenchyma, even when adjacent to an RCC
- IgG4-RD and RCC in different anatomic locations
- IgG4-RD detected only microscopically, invisible on imaging

No prior publication described a radiologically visible focal perinephric IgG4-RPF abutting an RCC and simulating extrarenal tumour extension.

7. Conclusion
Across all databases, no prior cases described a focal IgG4-related retroperitoneal fibrosis lesion immediately adjacent to a renal cell carcinoma and radiologically mimicking perinephric tumour extension (T4 disease).

Thus, this appears to be the first reported case of radiologically visible perinephric IgG4-related fibrosis directly adjacent to a renal cell carcinoma and mimicking its extrarenal extension, supporting the novelty of the case as of December 9, 2025.

8. Search Limitations

- Some queries were limited (Google Scholar screens up to 100 and Europe PMC A+B screens up to 750) because entries beyond this threshold were consistently duplicative or irrelevant.
- Searches were performed by a single reviewer due to the narrow scope of the query and the descriptive nature of novelty substantiation. However, this may introduce a minor risk of missed studies despite the broad search strategy.
- Some older case reports may exist in non-indexed regional journals not captured by the searched databases, though this is unlikely to affect the conclusion.
